# Mutations in *TSPEAR*, Encoding a Regulator of Notch Signaling, Affect Tooth and Hair Follicle Morphogenesis

**DOI:** 10.1371/journal.pgen.1006369

**Published:** 2016-10-13

**Authors:** Alon Peled, Ofer Sarig, Liat Samuelov, Marta Bertolini, Limor Ziv, Daphna Weissglas-Volkov, Marina Eskin-Schwartz, Christopher A. Adase, Natalia Malchin, Ron Bochner, Gilad Fainberg, Ilan Goldberg, Koji Sugawara, Avital Baniel, Daisuke Tsuruta, Chen Luxenburg, Noam Adir, Olivier Duverger, Maria Morasso, Stavit Shalev, Richard L. Gallo, Noam Shomron, Ralf Paus, Eli Sprecher

**Affiliations:** 1 Department of Dermatology, Tel Aviv Medical Center, Tel Aviv, Israel; 2 Department of Human Molecular Genetics and Biochemistry, Tel-Aviv University, Tel Aviv, Israel; 3 Division of Dermatology, University of California, San Diego, San Diego, California, United States of America; 4 Department of Dermatology, University of Münster, Münster, Germany; 5 Sheba Medical Center, Ramat Gan, Israel; 6 Department of Cell and Developmental Biology, Tel Aviv University, Tel Aviv, Israel; 7 Department of Dermatology, Osaka City University, Osaka, Japan; 8 Faculty of Chemistry, Technion, Haifa, Israel; 9 Laboratory of Skin Biology, National Institute of Health, Bethesda, Maryland, United States of America; 10 Institute of Human Genetics, Haemek Medical Center, Afula, Israel; 11 Rappaport Faculty of Medicine, Technion, Haifa, Israel; 12 Centre for Dermatology Research, Institute of Inflammation and Repair, University of Manchester, Manchester, United Kingdom; Thomas Jefferson University, UNITED STATES

## Abstract

Despite recent advances in our understanding of the pathogenesis of ectodermal dysplasias (EDs), the molecular basis of many of these disorders remains unknown. In the present study, we aimed at elucidating the genetic basis of a new form of ED featuring facial dysmorphism, scalp hypotrichosis and hypodontia. Using whole exome sequencing, we identified 2 frameshift and 2 missense mutations in *TSPEAR* segregating with the disease phenotype in 3 families. *TSPEAR* encodes the thrombospondin-type laminin G domain and EAR repeats (TSPEAR) protein, whose function is poorly understood. *TSPEAR* knock-down resulted in altered expression of genes known to be regulated by *NOTCH* and to be involved in murine hair and tooth development. Pathway analysis confirmed that down-regulation of *TSPEAR* in keratinocytes is likely to affect Notch signaling. Accordingly, using a luciferase-based reporter assay, we showed that *TSPEAR* knock-down is associated with decreased Notch signaling. In addition, NOTCH1 protein expression was reduced in patient scalp skin. Moreover, *TSPEAR* silencing in mouse hair follicle organ cultures was found to induce apoptosis in follicular epithelial cells, resulting in decreased hair bulb diameter. Collectively, these observations indicate that *TSPEAR* plays a critical, previously unrecognized role in human tooth and hair follicle morphogenesis through regulation of the Notch signaling pathway.

## Author Summary

Ectodermal dysplasias refer to a large group of inherited disorders characterized by developmental defects in tissues of ectodermal origin. The study of these conditions has been instrumental in the discovery of biological pathways involved in the regulation of epithelial tissue morphogenesis. In this report, through the delineation of the molecular basis of a novel form of autosomal recessive ectodermal dysplasia, we identified a new key player in ectodermal development. We detected a number of mutations in *TSPEAR* co-segregating with abnormal hair and tooth development in three families. *TSPEAR* encodes the thrombospondin-type laminin G domain and EAR repeats (TSPEAR) protein, whose function is poorly understood. TSPEAR was found to be strongly expressed in murine hair and tooth. Using a reporter assay, we showed that it regulates Notch activity. Accordingly, NOTCH1 expression was altered in patient skin, and NOTCH1, as well as many of its known targets, was down-regulated in TSPEAR deficient keratinocytes. Moreover, *Tspear* silencing in mouse hair follicle organ cultures was found to induce apoptosis in follicular epithelial cells, resulting in decreased hair bulb diameter. Collectively, these observations indicate that TSPEAR plays a critical, previously unrecognized role in human tooth and hair follicle morphogenesis through regulation of the Notch pathway. As such, these new data are likely to lead to further investigations aimed at characterizing the role of Notch signaling pathway in other forms of ectodermal dysplasias as well as acquired hair and tooth pathologies.

## Introduction

Ectodermal dysplasias refer to a large clinically and genetically heterogeneous group of disorders characterized by developmental defects affecting tissues of ectodermal origin [1]. These conditions therefore feature various combinations of cutaneous, nail, hair, dental or limb anomalies which demarcate the various subtypes of ED [1]. Over the past few years, the molecular basis of many of these diseases has been deciphered, leading to the identification of a number of signaling pathways responsible for regulating ectodermal tissue ontogenesis. Among these regulatory systems, the ectodysplasin/EDAR signaling pathway, which regulates NFkappaB activity and is critically involved in murine tooth and hair development [2], is the best known and was shown to be involved in the pathogenesis of various clinical forms of hypohidrotic ectodermal dysplasia [3,4]. Additional regulatory factors which were found to be involved in the pathogenesis of ectodermal dysplasias include p63, DLX3, MSX1 and WNT proteins [5–8]. Finally, structural proteins, such as connexins and desmosomal proteins, have also been implicated in the pathogenesis of a number of ectodermal dysplasias [9,10].

Although the concomitant presence of hair and tooth abnormalities is not unusual among ectodermal dysplasias, as seen in hypohidrotic ectodermal dysplasia (MIM305100) [11], neonatal ichthyosis-sclerosing cholangitis syndrome (MIM607626) [12], p63 syndromes [13] and alopecia-neurological defects-endocrinopathy (MIM612079) [14], it is rarely seen in the absence of other ectodermal or visceral defects. In the present study, we aimed at identifying the molecular basis of a novel form of ectodermal dysplasia combining scalp hypotrichosis and hypodontia.

## Results

### Clinical delineation of a novel form of ectodermal dysplasia

**Fig 1:**
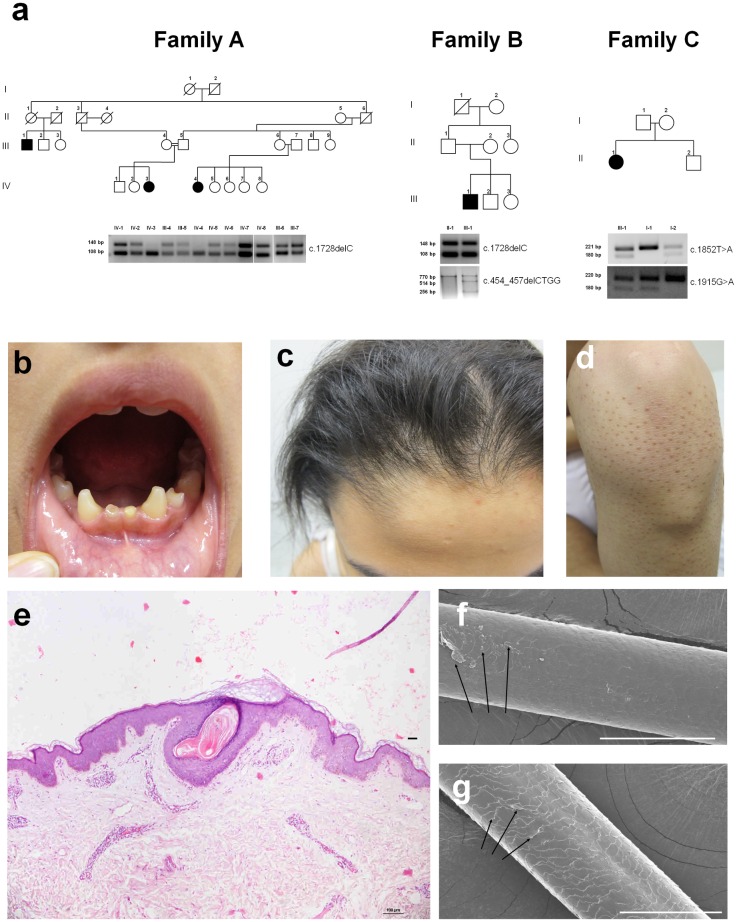
Clinical and pathological features. (a) Family pedigrees are presented in the upper panels. Black symbols denote affected individuals. PCR-RFLP assays, performed as detailed in materials and methods, were used in each family to confirm co-segregation of the mutation with the disease phenotype (lower panels). Mutation c.1728delC is associated with the presence of a 108 bp fragment in families A and B, while mutation c.454_457delCTGG results in a 514 and 256 bp fragments in family B; in addition, both mutations c.1852T>A and c.1915G>A are associated with the presence of a 180 bp fragment in family C; (b-d) Clinical features displayed by the patients include (b) hypodontia with conical teeth, (c) anterior scalp hypotrichosis and (d) follicular accentuation; (e) A skin biopsy obtained from scalp skin of individual IV-4 of family A and stained for hematoxylin and eosin, demonstrates paucity of rudimentary hair follicles; (f-g) Scanning electron microscopy (SEM) analysis of hair shafts obtained from the scalp demonstrates flattened and partially absent cuticular scales (arrows) in the patient hair (f) as compared with a healthy individual (g) (scale bar = 100 μm).

We studied two consanguineous families of Arab Moslem origin and one Jewish Ashkenazi family, comprising together a total of 5 patients (Fig 1A). All affected individuals displayed hypodontia (Fig 1B) as well as various degrees of scalp hypotrichosis more prominent on the anterior part of the scalp (Fig 1C). The severity of the phenotype, including the degree of alopecia and hypondontia was mild in family C patient. Patients also shared subtle dysmorphic features including a long oval face, square chin, down slanting of palpebral fissures, low insertion of columella and thick lips. We also identified in one patient (family A, IV-4) body hypertrichosis over the chest while in other patients of family A and B, body hair was missing or sparse. Follicular accentuation was most marked over bony prominences (Fig 1D). No visceral or neurological additional features were identified. Audiometry performed in family A and family C patients was normal (clinical information on all families is summarized in S1 Table). A skin biopsy obtained from patient IV-4 (Family A) scalp demonstrated paucity of mature hair follicles (Fig 1E). Scanning electron microscopy of patient hair samples showed abnormal structure of the follicular cuticle (Fig 1F).

### Mutation analysis

After having excluded by direct sequencing pathogenic mutations in the coding sequences of *WNT10A* and *TP63*, which have been associated with a phenotype reminiscent of that displayed by the patients [13,15], DNA samples extracted from individuals IV-4, III-7, IV-3 and III-5 of family A and individuals II-1, I-1 and I-2 of family C, were subjected to whole exome sequencing. Data were filtered as detailed in Materials and Methods and scrutinized for mutations in any single gene common to both families.

**Fig 2:**
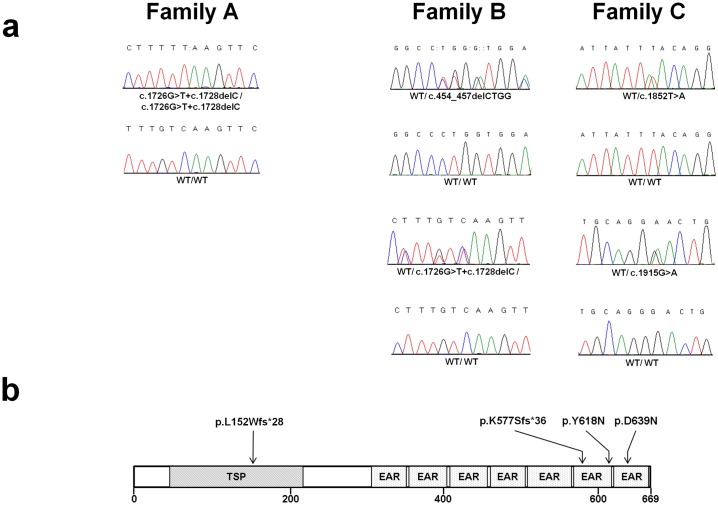
Mutation analysis. (a) Direct sequencing of *TSPEAR* revealed a homozygous missense transversion c.1726G>T and a homozygous c.1728delC deletion in family A patients; heterozygous c.454_457delCTGG, c.1726G>T and c.1728delC mutations in family B patient; and heterozygous c.1852T>A and c.1915G>A missense mutations in family C patient. Wildtype sequences are given below the mutant sequence for comparison; (b) The predicted consequences of the 4 mutations are depicted along a schematic representation of the TSPEAR protein structure with its different domains.

Using this approach, we identified three mutations in *TSPEAR* encoding thrombospondin-type laminin G domain and EAR repeats, a member of the EAR family of proteins [16]. These proteins feature EAR domains, which are likely to mediate protein-protein interactions [16]. All affected individuals of family A were found to carry a homozygous missense sequence variation, c.1726G>T, as well as a homozygous single base pair deletion in *TSPEAR*, c.1728delC (Fig 2A), whereas individual II-1 of family C carried two heterozygous missense mutations: c.1852T>A and c.1915G>A (Fig 2A). Individual III-1 of family B was subsequently found by direct sequencing of *TSPEAR* coding sequences to carry c.1726G>T in a heterozygous state as well as to be compound heterozygous for two heterozygous deletions, c.1728delC and c.454_457delCTGG (Fig 2A).

Mutations c.1728delC and c.454_457delCTGG are both predicted to result in premature termination of protein translation (p.K577Sfs*36; p.L152Wfs*28). Mutations c.1852T>A and c.1915G>A are expected to result in two amino acid substitutions, p.Y618N and p.D639N, respectively, affecting two highly conserved residues (Conseq scores 9 and 9, respectively; range 1–9) located in two EAR domains of the protein (Fig 2B). Both c.1852T>A and c.1915G>A were foreseen to be pathogenic by two prediction software (Polyphen2 scores 1 and 1, respectively; range 0–1; SIFT scores 0 and 0.04, respectively; range 1–0). Sequence variation c.1726G>T is likely to be in linkage disequilibrium with c.1728delC. c.1726G>T is predicted to result in a single amino acid substitution (p.V576F) whose significance is unclear given conflicting results of prediction software (damaging according to PolyPhen and tolerated according to SIFT). In addition, given the predicted effect of the adjacent frameshift c.1728delC, the functional consequence of p.V576F is likely to be marginal.

Co-segregation of all four mutations with the disease phenotype was then confirmed by PCR-RFLP (Fig 1A; see experimental details in Materials and Methods). Using the same assays, mutations c.1852T>A, c.1915G>A, c.454_457delCTGG and c.1728delC were excluded from a panel of 476, 415, 294 and 309 population-matched healthy individuals respectively. We then ascertained the ESP, NCBI, UCSC, HGMD, ExAc, 1000 genomes and Ensembl databases for the presence of each of the 4 mutations. Among these, mutation c.1915G>A was present in a heterozygous state in 0.7% of a panel of control individuals (n = 35,626), suggesting that it may be associated with a common phenotype in the general population such as hypondontia whose prevalence ranges between 2% and8% [17–19]. Mutation c.454_457delCTGG was absent in all public databases while mutation c.1852T>A was present in a heterozygous state in 2 individuals out of 60,136 tested. In addition, mutation c.1728delC was present in a heterozygous state in 3 individuals out of 60,032 tested.

### Functional characterization of *TSPEAR*

**Fig 3:**
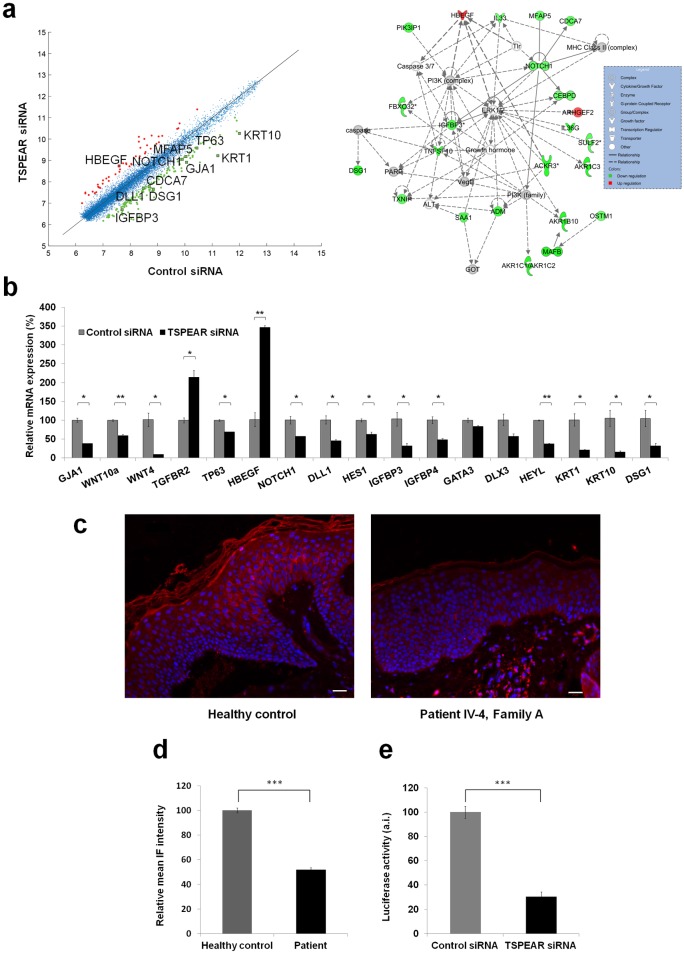
Expression analysis. (a) A comparative analysis of gene expression profiles of primary keratinocytes transfected with *TSPEAR* specific siRNA or control siRNA (left panel) revealed a number of differentially expressed genes including *NOTCH1* and additional genes encoding elements of the *NOTCH1* regulatory network and/or known to be involved in hair and tooth development. Pathway analysis (IPA software, see details in Materials and Methods, right panel) revealed that *TSPEAR* down-regulation affects a *NOTCH*-associated regulatory network; (b) Gene expression following siRNA-mediated *TSPEAR* down-regulation was assessed using qRT-PCR. Results are expressed as percentage of gene expression in cells transfected with *TSPEAR*-specific siRNA relative to gene expression in siRNA control-transfected cells ± standard error (two sided t-test; *p<0.05, **p<0.01). Results are normalized to *GAPDH* RNA levels; (c,d) NOTCH1 expression was assessed by immunostaining (c) in skin biopsies obtained from an affected individual (IV-4, family A; right panel) and from a healthy individual (left panel). Immunostaining was significantly reduced in affected vs. normal skin (d) (scale bars = 25 μm; (e) HaCaT cells were co-transfected with a NOTCH1-responsive luciferase reporter gene and *TSPEAR*-specific siRNA or control siRNA. Luciferase activity was measured after 48 hours and normalized to Renilla luciferase. Results represent the mean of three independent experiments ± standard error (two sided t-test; ***p<0.001).

Given the phenotype displayed by patients carrying biallellic mutations in *TSPEAR* and because the role of TSPEAR in cutaneous tissues is unknown [16,20], we hypothesized that *TSPEAR* may be involved in the regulation of human tooth and hair follicle morphogenesis. To explore this hypothesis, we used microarray analysis to compare whole exome expression profiles of primary human keratinocytes transfected with control or *TSPEAR*-specific siRNA (see experimental details in Materials and Methods and S1 Fig). Pathway analysis of the data (fully available in S2 Table) revealed down-regulation of *NOTCH1* as well as abnormal expression of numerous Notch signaling pathway-associated genes (Fig 3A and S3 Table). Quantitative RT-PCR was used to validate these observations (Fig 3B). Interestingly, immunostaining of a skin biopsy obtained from patient IV-4, family A, also revealed decreased NOTCH1 expression in the epidermis (Fig 3C and 3D), thus supporting the hypothesis that *TSPEAR* mutations exert a loss of function effect mediated through *NOTCH1*.

To ascertain the possibility that TSPEAR regulates ectodermal ontogenesis by modulating Notch signaling, we co-transfected HaCaT cells seeded on DLL1-coated plate with a Notch luciferase reporter construct and with a *TSPEAR*-specific siRNA or a control siRNA. Luciferase activity in TSPEAR down-regulated cells was significantly decreased as compared with control cells, supporting a role for TSPEAR in the regulation of Notch signaling (Fig 3E).

**Fig 4:**
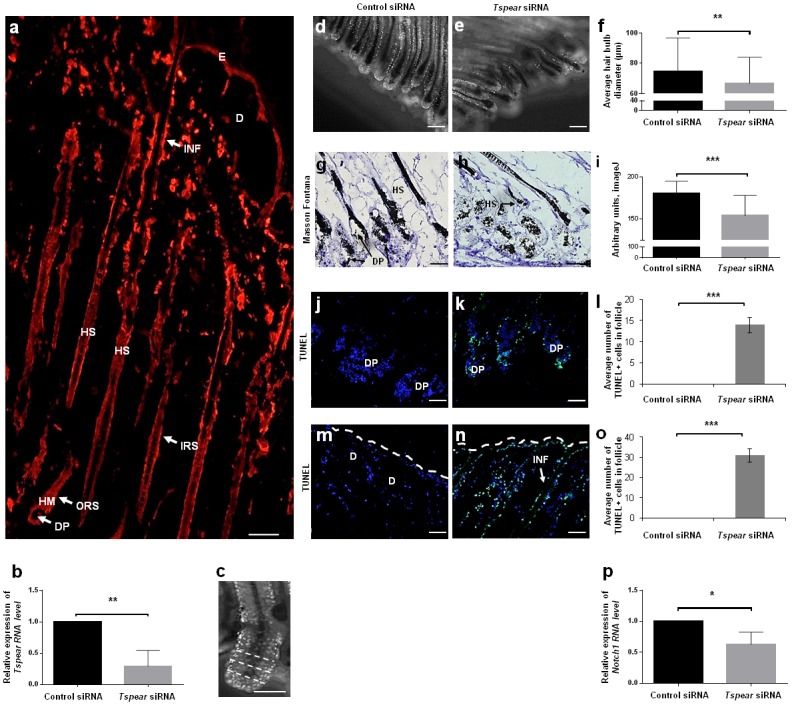
Effect of *Tspear* down-regulation on murine hair follicles. (a) Tspear is expressed in mouse hair follicles (HFs) in the hair matrix keratinocytes, outer root sheath, inner root sheath, hair shaft and the infundibulum (scale bar = 50 μm); (b) Back skin tissue strips from *K14-H2B-GFP* mice were transfected with *Tspear* siRNA or control siRNA. RNA was extracted from transfected HFs and *Tspear* RNA expression levels were assessed by qRT-PCR. Results were normalized to *Gapdh* levels and are expressed as expression levels relative to control samples. Data were pooled from three independent experiments (two sided t-test; **p<0.01); (c-f) Z stacks optical sections of *K14-H2B-GFP* mouse HFs (c) obtained 24h following transfection with control siRNA (d) or *Tspear* siRNA (e) were used to calculate average hair bulb diameter. Three measurements were done for each HF in the bulb and proximal hair shaft (c, dashed white lines) and an average diameter was calculated accordingly. Epithelial nuclei are marked with *GFP* (scale bars = 100 μm). Data was pooled from three independent experiments (F, two sided t-test; **p<0.01); (g-i) Melanin content was assessed by quantitative Masson-Fontana histochemistry in *Tspear* siRNA treated HFs (h) compared to control (g). Data was pooled from two independent experiments (I, two sided t-test; ***p<0.001) (scale bars = 50 μm); (j-o) Apoptosis was assessed by the TUNEL assay (TUNEL, green; DAPI, blue) at the hair bulb (j-l) and infundibular (m-o) compartments of HFs downregulated for *Tspear* (k,n) compared to control siRNA treated HFs (j,m) (scale bars = 50 μm). Average number of TUNEL-positive cells in hair follicles in the respective compartments. Data were pooled from two independent experiments (l,o, two sided t-test; ***p<0.001) (scale bars = 50 μm). White dotted lines delineate the outer epidermal surface; (p) RNA was extracted from *Tspear* siRNA and control siRNA transfected HFs and *Notch1* RNA expression level was assessed by qRT-PCR. Results were normalized to *Rplp0* levels and are expressed as expression levels relative to control samples. Data were pooled from three independent experiments (two sided t-test; *p<0.05). E—epidermis; INF–Infundibulum; D—dermis; DP—dermal papilla; IRS—inner root sheath; ORS—outer root sheath; HM—hair matrix; HS—hair shaft; TUNEL—terminal deoxynucleotidyl transferase dUTP nick end labeling.

*NOTCH1* has been associated with the regulation of dental epithelial stem cells differentiation [21] and TSPEAR was found to be expressed in the enamel organ (S2 Fig). In addition, NOTCH1 is essential for normal hair follicle postnatal development [22–26]. To investigate the role of TSPEAR in hair follicles, we obtained skin biopsies from transgenic *K14/H2B/GFP* mice which express green fluorescent protein (GFP) in hair follicle epithelium (see experimental details in Materials and Methods). Tspear was found to be expressed in murine hair matrix keratinocytes, outer root sheath, inner root sheath, hair shaft and the hair follicle infundibulum (Fig 4A). We then down-regulated *Tspear* expression using specific siRNAs. siRNA-mediated down-regulation of *Tspear* in mouse skin organ cultures (Fig 4B) resulted in reduced hair bulb diameter (Fig 4C–4F). This correlated with hair growth arrest as attested by decreased hair follicle pigmentation (Fig 4G–4I) (HF pigmentation is closely linked to the growth phase of the hair cycle (anagen) [27], and markedly elevated apoptotic activity both in the hair bulb (Fig 4J–4L) and infundibular (Fig 4M–4O) hair follicle compartments, as measured by the TUNEL assay. *Tspear* knock-down also resulted in decreased *Notch1* expression in murine hair follicle organ cultures (Fig 4P).

## Discussion

In the present report, we studied a novel form of ectodermal dysplasia characterized by oligodontia, alopecia and facial dysmorphism and caused by mutations in *TSPEAR*. The physiological functions of TSPEAR are essentially unknown to date. It belongs to a family of proteins featuring EAR domains, which are predicted to form beta-propeller structures likely to mediate protein-protein interactions [16]. Some of these proteins have been found to be associated with various neurological conditions [16]. Mutation c.1728delC in *TSPEAR* has been reported to cause congenital sensorineural deafness in a single family and to result in inhibition of TSPEAR secretion [28]. However, this mutation was identified in the present study in a homozygous state in 2 different patients with hypotrichosis, hypodontia and normal hearing (Fig 1A). This observation coupled with the fact that we identified three other mutations in *TPSEAR* in additional patients with ectodermal dysplasia and normal hearing suggests the possibility that deafness in this previous single family [28] may have been due to co-inheritance of additional genetic variants. In contrast, an association study recently demonstrated the presence of genome-wide significant variations within the *TSPEAR* gene locus for sheep fiber diameter[29], which is in line with the reduced hair bulb diameter caused by *TSPEAR* down-regulation in mouse hair follicles (Fig 4F).

Although the detailed mechanism of action of *TSPEAR* during tooth and hair follicle morphogenesis remains to be fully delineated, our current data support the possibility that *TSPEAR* regulates Notch signaling, a key biological pathway previously shown to affect the development of many ectodermal tissues [23,30,31]. Although decreased Notch expression due to nicastrin mutations has been associated with perifollicular inflammation [32], overt inflammatory manifestations were not observed in patients carrying TSPEAR mutations possibly due to the fact nicastrin may affect additional targets beyond Notch and/or Notch signaling may be affected to a lesser degree by *TSPEAR* mutations as compared with nicastrin mutations. Supporting this possibility is the presence of follicular accentuation in our patients (Fig 1D). Interestingly, a number of known targets of *NOTCH* which were found to be affected by *TSPEAR* silencing in the current study (Fig 3B), have previously been associated with disorders featuring abnormal hair and tooth development including the oculo-dento-digital dysplasia syndrome (MIM257850), the odonto-onycho-dermal dysplasia syndrome (MIM257980), the p63 syndromes (MIM604292) and the tricho-dento-osseous syndrome (MIM190320) caused by mutations in *GJA1*, *WNT10A*, *TP63 and DLX3*, respectively [13,15,33–35]. Collectively, these data demarcate a group of inherited disorders sharing both phenotypic and pathophysiological features.

## Materials and Methods

### Patients

All affected and healthy family members or their legal guardian provided written and informed consent according to a protocol approved by our institutional review board and by the Israel National Committee for Human Genetic Studies in adherence with the Helsinki principles.

### DNA extraction

Genomic DNA was extracted from peripheral blood leukocytes using the 5 Prime ArchivePure DNA Blood Kit (5 Prime Inc., Gaithersburg, USA) or from OG-500 saliva collection kit (DNA Genotek Inc., Ottawa, Canada) according to the manufacturer's instructions.

### Exome sequencing

Exome sequencing of individuals IV-3, IV-4, III-5, III-7 from family A, I-1, I-2 and II-1 from family C was performed by Otogenetics corporation using in-solution hybridization with Agilent AV5 + UTR Exome (71Mb) version 4.0 (Agilent, Santa Clara, USA) followed by massively parallel sequencing (Illumina HiSeq2000) with 100-bp paired-end reads. Reads were aligned to the Genome Reference Consortium Human Build 37 (GRCh37/hg19) using Burrows-Wheeler Aligner (BWA)[36].

Duplicate reads, resulting from PCR clonality or optical duplicates, and reads mapping to multiple locations were excluded from downstream analysis. Reads mapping to a region of known or detected insertions or deletions were re-aligned to minimize alignment errors. Single-nucleotide substitutions and small insertion deletions were identified and quality filtered using the Genome Analysis Tool Kit (GATK) [37]. Rare variants were annotated using ANNOVAR[38] and identified by filtering the data from dbSNP138, the 1000 Genomes Project, the Exome Variant Server, and an in-house database of sequenced individuals. Variants were classified by predicted protein effects using Polyphen2 [39] and SIFT [40]. S4 Table summarizes exome sequencing details.

### Mutation analysis

Genomic DNA was PCR-amplified using oligonucleotide primer pairs spanning the entire coding sequence as well as intron–exon boundaries of *WNT10A*, *TP63* and *TSPEAR* (S5 Table) and Taq polymerase (Qiagen, Hilden, Germany). Cycling conditions were as follows: 94°C, 2min; 94°C, 40 sec; 61°C, 40 sec; 72°C 50 sec, for 3 cycles, 94°C, 40 sec; 59°C, 40 sec; 72°C 50 sec, for 3 cycles, 94°C, 40 sec; 57°C, 40 sec; 72°C 50 sec, for 34 cycles. Gel-purified (QIAquick gel extraction kit, QIAGEN, Hilden, Germany) amplicons were subjected to bidirectional DNA sequencing with the BigDye terminator system on an ABI Prism 3100 sequencer (Applied Biosystems, NY, USA).

### PCR-restriction fragment length polymorphism (RFLP)

To screen for the c.1728delC mutation (families A and B), we PCR-amplified a 148 bp fragment with Taq polymerase (Qiagen, Hilden, Germany) and the following primers 5`- CTCCGTCATCTACGAGCTGAACGTGACCGCGCAGGCCTTTTT-3`and 5`- GATGAGCCTAACGGGGATTCC-3`. The mutation creates a recognition site for endonuclease MseI (New England Biolabs, Frankfurt, Germany). To screen for the c.454_457delCTGG mutation (family B), we PCR-amplified a 770 bp fragment, with Taq polymerase (Qiagen, Hilden, Germany) and the following primers 5`- TCTCACCACCTGTGCTCATC-3`and 5`- CACCTGTTCTCGCCAATGTC -3`. The mutation creates a recognition site for endonuclease BglI (New England Biolabs, Frankfurt, Germany). To screen for the c.1852T>A mutation (family C), we PCR-amplified a 221 bp fragment, with Taq polymerase (Qiagen, Hilden, Germany) and the following primers 5`- GTAGCTTCTGGCCAATCCCC-3`and 5`- GAAGCAAG GCTCTGGGAGG-3`. The mutation creates a recognition site for endonuclease MseI (New England Biolabs, Frankfurt, Germany). To screen for the c.1915G>A mutation (family C), we PCR-amplified a 220 bp fragment, with Taq polymerase (Qiagen, Hilden, Germany) and the following primers 5`- GGATGGAAGAGGCTCAGATG-3`and 5`- AGATGAGG TAGGCACCAGCCGTGGTGCTGAAGGCCTCCGAAT-3`. The mutation creates a recognition site for endonuclease EcoRI (New England Biolabs, Frankfurt, Germany). PCR cycling conditions were as follows: 94°C, 2min; 94°C, 40 sec; 61°C, 40 sec; 72°C 50 sec, for 3 cycles, 94°C, 40 sec; 59°C, 40 sec; 72°C 50 sec, for 3 cycles, 94°C, 40 sec; 57°C, 40 sec; 72°C 50 sec, for 34 cycles. PCR products were incubated with the appropriate enzyme at 37°C for 16 hours followed by 20 min of inactivation at 65°C. The digested PCR products were electrophoresed in ethidium bromide-stained 3% agarose gels.

### Quantitative RT-PCR

For quantitative real-time PCR (qRT-PCR), cDNA was synthesized from 1000 ng of total RNA using qScript kit (Quanta Biosciences, Gaithersburg, MD). cDNA PCR amplification for *TSPEAR* and *GAPDH* (as a control) was performed with TaqMan SNP expression assays # Hs00376562_m1 and Hs02758991_g1 respectively (Applied Biosystems, Forster, CA, USA) according to the manufacturer protocol. cDNA PCR amplification for other genes was carried out with the PerfeCTa SYBR Green FastMix (Quanta Biosciences, Gaithersburg, USA) on a StepOnePlus system (Applied Biosystems, Waltham, USA) with gene-specific intron-crossing oligonucleotide pairs (S6 Table). Cycling conditions were as follows: 95°C, 20 sec and then 95°C, 3 sec; 60°C, 30 sec for 40 cycles. Each sample was analyzed in triplicates. For each set of primers, standard curves were obtained with serially diluted cDNAs. Results were normalized to *GAPDH* mRNA levels. qRT-PCR results were analyzed by *t-test* statistical analysis.

### Cell cultures

Keratinocytes (KCs) cell cultures were established from skin biopsies after written informed consent had been obtained as previously described [41]. Primary KCs were maintained in KC Growth Medium (KGM) supplemented with 0.4% bovine pituitary extract, 0.1% human epidermal growth factor (hEGF), 0.1% insulin, 0.1% hydrocortisone and 0.1% gentamicin/amphotericin B. HaCaT cells were kindly provided by Dr. Dina Ron (Technion, Haifa, Israel). The cells were maintained in MEM media supplemented with 10% fetal calf serum, 1% L-glutamine, 1% streptomycin and 1% amphotericin (Biological Industries, Beit-Haemek, Israel).

### siRNA transfection

KCs were cultured in 6 well culture plates at 37°C in 5% CO_2_ in a humidified incubator and were harvested at 60% confluence. To down regulate *TSPEAR* expression, we used human TSPEAR small interference RNAs (siRNA) (Santa Cruz; sc-62060) (5`-CCUUCUCGGUGAACAGUAUtt-3`, 5`-CAUUGCCGC CACCUAUUUAtt-3`and 5`-CACUCCUGACCUUUCGUAAtt-3`). As control siRNA, we used Stealth RNAi Negative Control Duplex (Invitrogen, Carlsbad, CA). Twenty five pmol of siRNAs were transfected into KCs using Lipofectamine RNAiMax (Invitrogen). The transfection medium was replaced after 6 hours with high calcium (1.4mM)-containing KGM.

### Gene expression microarray and pathway analysis

Total RNA (200 ng) was reverse transcribed and cRNA prepared using TargetAmp-Nano Labeling Kit (Epicentre Biotechnologies, Madison, WI) according to the manufacturer's protocol. One and a half μg of biotinylated cRNA was hybridized to HumanHT-12 v4 Expression BeadChip (encompassing more than 47,000 transcript targets), washed, and scanned on a BeadArray 500GX Reader using Illumina BeadScan image data acquisition software (version 2.3.0.13). Quality control and quantile normalization of the microarray data was done by BeadStudio 3.0 software (Illumina). The scanning data of the three biological repeats (total of 12 data sets) were exported to JMP genomic Software (SAS, Cary, NC), log transformed and non-expressed genes (detection p-value<0.01), transcripts with low expression (log2 value < 6.5) or with low variation across all samples (variation < 0.05) were removed from the analysis. The data was analyzed using two-way ANOVA and differently expressed genes (DEGs) were defined as transcripts that were statistically significant at corrected p-value ≤0.05 using the False Discovery Rate (FDR) with at least 0.75 delta differences. Pathway analysis to identify statistically significant functional categories in the data set was performed using Ingenuity Pathway analysis (IPA 8.0, QIAGEN Redwood City, www.qiagen.com/ingenuity).

### Immunostaining

For immunofluorescence analysis of skin biopsies, 5 μm paraffin-embedded sections were kept overnight at 37°C and de-paraffinized using xylene/ethanol. Antigen retrieval was done with 0.01M citrate buffer, pH 6.0 (Invitrogen, Carlsbad, CA) in a microwave for 25 min. Sections were blocked with 2% bovine serum albumin (BSA) in phosphate-buffered saline (PBS) for 30 min at room temperature. Primary antibodies used: rabbit anti-TSPEAR primary antibody (Abcam, Cambridge, MA, USA, 1:200 dilution); goat anti-NOTCH primary antibody (Santa Cruz, Dallas, TX, USA, 1:75 dilution). Both antibodies were diluted in 2% BSA PBS and incubated overnight at 4°C. Rhodamine Red-X goat anti rabbit IgG (H+L) (Life Technologies/Invitrogen) and Alexa Fluor 568 donkey anti goat IgG (H+L) (Thermo Fisher Scientific) were used as a secondary antibody and were diluted 1:200 with 2% BSA in PBS followed by incubation for 45 min at room temperature. Coverslips were mounted in DAPI Fluoromount-G (Southern Biotechnologies, Birmingham, AL). Negative controls consisted of slides processed similarly while omitting the primary antibody. As a positive control for TSPEAR staining, we used normal placenta tissue[42]. Specimens were examined using either a Nikon 50I microscope connected to DS-RI1 digital camera or a Zeiss LSM700 confocal microscope for fluorescence image acquisition.

### NOTCH1 reporter assay

HaCaT cells were seeded on hDLL1 (R&D Systems, Minneapolis, MN) coated 24 wells plate (50,000 cells/well). Twenty four hours after seeding, cells were transfected with a Notch response element-containing luciferase reporter construct, kindly obtained from Dr. David Sprinzak (Biochemistry Department, The George S. Wise Faculty of Life Sciences, Tel Aviv University) as well as a Renilla expression vector, and control siRNA (Stealth^™^ RNAi Negative Control Duplex Invitrogen, Carlsbad, CA) or TSPEAR specific siRNA (sc-91435; Santa Cruz Biotechnology, Santa Cruz, CA) using Lipofectamine2000 (Invitrogen, Carlsbad, CA). Forty eight hours after transfection, luciferase activity was read using a dual luciferase assay (Promega, Madison, USA). Luciferase activity was normalized to Renilla luciferase.

### *Tspear* knockdown in mice hair follicles

*K14-Cre* and *H2B-GFP lox*P mice were purchased from The Jackson Laboratory. *K14-Cre* mice contain a human keratin 14 promoter directing expression of Cre recombinase, while *H2B-GFP* mice have a fusion H2B histone with a C-terminally attached eGFP. *K14-Cre* and *H2B-GFP lox*P mice were crossed, heterozygous littermates interbred, and resulting pups genotyped. Homozygous *K14 H2B-GFP*^*+/+*^ mice were selected for and used as breeding pairs. All mice were kept at the University of California, San Diego (UCSD) animal facilities, and all animal experiments were approved by the UCSD Institutional Animal Care and Use Committees and were conducted in accordance with the Guideline for the Care and Use of Laboratory Animals.

Dorsal skin was isolated from *K14-H2B-GFP* mice and placed dermal side down into individual sterile petri dishes containing 4 ml warmed supplemented William’s E. Media (WEM) as previously described[43], with the orientation of the hair parallel to the *longitudinal* axis. Thin dorsal tissue strips were then sliced off, gently abrading to remove any loose hair.

Tissue strips were transfected with mouse *Tspear* siRNA (sc-270602; Santa Cruz Biotechnology, Santa Cruz, CA) or control siRNA (sc-36869; Santa Cruz Biotechnology). All reagents required for transfection were obtained from Santa Cruz Biotechnology (siRNA transfection reagent, sc-29528; siRNA transfection medium, sc-36868). Transfection was performed as previously described [43] and following 7 hours of transfection, tissue strips were maintained in 6-well plate with 2 ml supplemented WEM for an additional 24 hours. Following 24 hours, two tissue strips from each treatment group were used for average hair bulb measurement. In detail, two hundred μl of 2% low melt agarose solution (Agarose II; Midsci, MO, USA) dissolved in sterile Dulbecco’s phosphate buffered saline (DPBS) were used to coat the bottom of 6-well glass bottom plate (MatTek, Ashland, MA). Dorsal tissue strips were positioned parallel to the bottom of the plate, and embedded in agar. One ml of WEM was gently added to the wells. The 6-well plate was then placed in a pre-warmed incubation chamber (37°C, 5% CO_2_) of a Zeiss Axio Observer.Z1 microscope. Axiovision software (4.8.2 SP3) was used to select and mark multiple non-overlapping fields of view on each tissue strip covering a significant area of each strip. Fluorescent images were captured using an automatic exposure time and an excitation wavelength of 470 nm. Data were analyzed in a single blinded manner. Raw data collected were given an alpha-numeric cipher and were subsequently analyzed by a blinded investigator unaware of the conditions tested or grouping. In each hair follicle, three horizontal measurements were done in the area of hair bulb and proximal hair shaft. The average of those three measurements was calculated for each hair follicle from the two different treatment group and was defined as average hair bulb diameter. Three independent experiments were done with 3 different mice. In each mouse, 2 skin samples from each of the two treatment groups (*Tspear*-siRNA vs. control-siRNA) were used for average hair bulb measurement.

In addition, 3 strips from each treatment group were collected 24 hours following transfection for RNA isolation (RNeasy kit, Qiagen, Valencia, CA) and validation of *Tspear* silencing. For reverse transcription, we used an iScript cDNA synthesis kit (Bio-Rad, Hercules, CA) with 1 μg of RNA as starting material. The resulting cDNA was diluted 1:10 with nuclease free water and 5 μl of the diluted solution was used as template for subsequent qPCR reactions.

Quantitative real-time PCR was performed on an Applied Biosystems 7300 using TaqMan Universal PCR Master Mix (Applied Biosystems, Carlsbad, CA), template cDNA, and TaqMan primers. TaqMan primers were ordered from Life technologies; *Tspear* (Mm00455327_m1), and *Gapdh* (Mm99999915_g1) which served to normalize data (Life Technologies/Invitrogen, Grand Island, NY). To quantify *Notch1* expression, we used 2x SYBR Green qPCR Master Mix (Biotool, Houston,TX) and Primer bank ID 13177625a1 *Notch1* primers while *Rplp0* served as an internal control (Forward primer 5’-GAGATTCGGGATATGCTGTTGG-3’ and Reverse primer 5’-CGGGTCCTAGACCAGTGTTCT-3’).

Finally, tissue strips from each treatment group were frozen in liquid nitrogen 48 hours after transfection for immunohistochemistry studies. Seven μm-thick cryosections were prepared and stored at −80°C until use. For Tspear protein expression, cryosections were first air-dried for 10 min and then fixed in acetone at -20°C for another 10 min. After air drying, the slides were washed three times for 5 min in PBS. Following 20 min incubation of cryosections with 2% goat serum in PBS, cryosections were incubated overnight at 4°C with the rabbit anti-Tspear monoclonal antibody (Abcam, Cambridge, MA) at 1:50 dilution with 2% goat serum in PBS solution. This was followed by incubation with Alexa Fluor^®^ 568 Goat Anti-Rabbit secondary Antibody (Life Technologies/Invitrogen) for 45 min at RT in 1:200 dilution with 2% goat serum in PBS solution. Incubation steps were interspersed with three washes, 5 min each, with PBS. Then sections were embedded and counterstained with DAPI for the identification of cell nuclei.

For Masson-Fontana histochemistry, cryosections were air dried and fixed in ethanol-acetic acid. The sections were washed in Tris-buffered saline (TBS) and distilled water several times. Cryosections were treated with ammoniacal silver solution (Thermo Fisher Scientific, Carlsbad, CA) for 40 min at 56°C in the dark. After washing in distilled water, the sections were treated with 5% aqueous sodium thiosulphate (Sigma-Aldrich, St. Louis, MO) for 1 min. Then, the sections were washed in running tap water for 3 min and were counterstained with haematoxylin for 45 seconds. After washing in distilled water, sections were dehydrated and mounted in Eukitt (Sigma-Aldrich, St. Louis, MO). Tspear immunoreactivity and melanin content by Masson-Fontana histochemistry were compared between test and control sections by quantitative histomorphometry as previously described [43,44] using NIH IMAGE software (NIH, Bethesda, MD, USA).

For apoptosis detection, a kit for DeadEnd Fluorometric TUNEL system analysis (Promega, Madison, WI, USA) was used. Briefly, cryosections were air dried and fixed in 4% formaldehyde in PBS for 25 minutes. Following several washing steps in PBS, permeabilization step with 0.2% Triton in PBS was conducted for 5 min. Following several washing steps in PBS, sections were equilibrated with equilibration buffer for 5–10 min and then incubated with TdT reaction mix for 60 minutes at 37°C. Following stop reaction step and washing steps with PBS, sections were embedded and counterstained with DAPI for the identification of cell nuclei.

### Electronic databases

The URLs for data presented herein are as follows:

1000 genomes project, http://www.1000genomes.org/

ConSurf, http://consurftest.tau.ac.il/

dbSNP, http://www.ncbi.nlm.nih.gov/SNP/

Exome Variant Server (http://evs.gs.washington.edu/EVS/)

GenBank, http://www.ncbi.nlm.nih.gov/Genbank/

NHLBI Grand Opportunity Exome Sequencing Project, https://esp.gs.washington.edu/drupal/

Online Mendelian Inheritance in Man (OMIM), http://www.omim.org

PolyPhen-2, http://genetics.bwh.harvard.edu/pph2/

SIFT, http://sift.jcvi.org/

UCSC Genome Browser, http://genome.ucsc.edu/

Human Gene Mutation Database, http://www.hgmd.cf.ac.uk/ac/index.php

ANNOVAR, http://annovar.openbioinformatics.org/en/latest/

Burrows-Wheeler Aligner, http://bio-bwa.sourceforge.net/

GATK, https://www.broadinstitute.org/gatk/

Ingenuity Pathway Analysis (IPA), www.qiagen.com/ingenuity

## Supporting Information

S1 Table Clinical features of ectodermal dysplasia in three families. Abbreviations: F, female; M, male; N, normal, not known; ^a^Body hypertrichosis in patient IV-3; ^b^hypotrichosis in lower limbs; ^c^during childhood.
(DOCX)

S2 Table Effect of *TSPEAR* down-regulation in keratinocytes on gene expression. (DOCX)

S3 Table Ingenuity Pathway Analysis (IPA) of upstream regulators. (Global gene expression data from human primary KCs transfected with control siRNA or *TSPEAR* siRNA (pooled from three independent experiments) were analyzed for upstream regulators using the IPA software).
(DOCX)

S4 Table Exome sequencing data. (DOCX)

S5 Table Oligonucleotide sequences used for DNA sequencing. (DOCX)

S6 Table Oligonucleotide sequences used for qRT-PCR. (DOCX)

S1 Fig TSPEAR mRNA expression in siRNA treated keratinocytes. *TSPEAR* mRNA expression in human primary KCs transfected with control siRNA or *TSPEAR* siRNA was ascertained using qRT-PCR. Results are expressed as percentage of gene expression in primary KCs cells transfected with *TSPEAR*-specific siRNA relative to gene expression in siRNA control-transfected cells ± standard error (two sided t-test: **p<0.01). Results are normalized to *GAPDH* RNA levels.
(DOCX)

S2 Fig *Tspear* expression in the enamel organ. (a) TSPEAR mRNA expression was assessed in mouse enamel organ. RNA was isolated from the enamel organ of four mice at postnatal day 10 and analyzed by RNA-seq. RNA-seq signal files were loaded into IGV (mm9) for peak visualization. The IGV track in the upper panel shows the peaks corresponding to exons 4 to 7 of Tspear. Note that Tspear is not annotated in IGV. Therefore we aligned the track to the corresponding region in UCSC genome browser (lower panel) where Tspear is annotated; (b) Immununohistochemical staining of longitudinal sections of rat mandibular incisors at P10 using anti-KRT5 (red) and anti-TSPEAR (green) antibodies. Nuclei are stained with DAPI (blue). TSPEAR was detected at the secretion front of ameloblasts at the secretory stage and persisted in the enamel matrix during the early maturation stages (white arrowheads). No staining was not detected at the late maturation stage. KRT5 was detected both in ameloblasts (above the white dotted lines) and in the papillary layer (below the white dotted lines).
(DOCX)
