# Supplementary material for: Mutations in TSPEAR, Encoding a Regulator of Notch Signaling, Affect Tooth and Hair Follicle Morphogenesis
Source: PLoS Genet. 2016 Oct 13;12(10):e1006369. doi: 10.1371/journal.pgen.1006369 (PMC5065119; doi:10.1371/journal.pgen.1006369)
Supplement: S1 Table — Abbreviations: F, female; M, male; N, normal, not known; aBody hypertrichosis in patient IV-3; bhypotrichosis in lower limbs; cduring childhood. (DOCX) [file pgen.1006369.s001.docx]

**S1 Table. Clinical features of ectodermal dysplasia in three families**

| Family | Scalp hypotrichosis | Body Hair | Hypodontia and conical teeth | Decreased sweating | Facial dysmorphism |
| --- | --- | --- | --- | --- | --- |
| A | ++/+++ | -^a^ | + | + | + |
| B | ++ | -^b^ | + | + | + |
| C | + | N | + | - | + |

Abbreviations: F, female; M, male; N, normal, not known; ^a^Body hypertrichosis in patient IV-4; ^b^hypotrichosis in lower limbs; ^c^during childhood.
