# Supplementary material for: Mutations in TSPEAR, Encoding a Regulator of Notch Signaling, Affect Tooth and Hair Follicle Morphogenesis
Source: PLoS Genet. 2016 Oct 13;12(10):e1006369. doi: 10.1371/journal.pgen.1006369 (PMC5065119; doi:10.1371/journal.pgen.1006369)
Supplement: S2 Table — (DOCX) [file pgen.1006369.s002.docx]

**S2 Table. Effect of TSPEAR down-regulation in KCs on gene expression**

| **Gene name** | **Fold-Change** | **P value** |
| --- | --- | --- |
| MT1G | 3.468 | 9.00424E-05 |
| KRT19 | 2.954 | 0.001031292 |
| LOC646723 | 2.808 | 0.003773244 |
| KRT8 | 2.616 | 0.000869668 |
| LOC644743 | 2.333 | 0.010240645 |
| LOC728285 | 2.222 | 0.001308602 |
| HBEGF | 2.212 | 0.00403349 |
| ANGPTL4 | 2.199 | 0.003862279 |
| TGM2 | 2.160 | 0.00067859 |
| FLJ40504 | 2.124 | 0.004134773 |
| KRT18P13 | 2.110 | 0.004190262 |
| ARL6IP1 | 2.105 | 0.00335803 |
| DDAH1 | 2.075 | 0.002828948 |
| RAB8B | 2.021 | 0.000662331 |
| STS-1 | 2.012 | 0.001120929 |
| RASA1 | 1.972 | 0.000258477 |
| XDH | 1.961 | 0.013698447 |
| MTHFD2 | 1.950 | 0.000338436 |
| GPD1L | 1.945 | 0.000274123 |
| SLC20A1 | 1.921 | 0.036927767 |
| CAMK2N1 | 1.905 | 0.002250057 |
| HS3ST1 | 1.894 | 0.022890845 |
| LOC647954 | 1.889 | 0.048349102 |
| STC2 | 1.883 | 0.027514329 |
| LOC149501 | 1.872 | 0.018132477 |
| MTHFD2 | 1.856 | 0.000227185 |
| CLDN7 | 1.830 | 0.015514254 |
| MT1F | 1.814 | 0.000465239 |
| MUC1 | 1.812 | 0.000192327 |
| NT5E | 1.766 | 0.03937834 |
| LEMD1 | 1.766 | 0.002033198 |
| SFXN4 | 1.765 | 7.34837E-05 |
| ARHGEF2 | 1.749 | 0.015447643 |
| COPS8 | 1.741 | 0.008664469 |
| ASAP2 | 1.732 | 0.013765664 |
| ADAM8 | 1.712 | 0.004112866 |
| FEZ2 | 1.701 | 0.018863102 |
| KRT81 | 1.701 | 5.20394E-05 |
| ADAM19 | 1.700 | 0.004594874 |
| RAC2 | 1.696 | 0.004499418 |
| TMEM16A | 1.696 | 0.028685884 |
| PRKCDBP | 1.684 | 0.012742037 |
| DCBLD2 | 1.682 | 0.019861301 |
| SWAP70 | -1.682 | 0.009843249 |
| RAB7B | -1.684 | 0.003023471 |
| HERC5 | -1.686 | 0.005193335 |
| MLLT11 | -1.686 | 0.005119317 |
| NAV2 | -1.689 | 0.00632364 |
| DSC2 | -1.699 | 0.002457376 |
| SULF2 | -1.699 | 0.025279656 |
| LOC645638 | -1.707 | 0.000490309 |
| SULF2 | -1.709 | 0.01301726 |
| CCNA2 | -1.710 | 0.022775072 |
| MXRA5 | -1.712 | 0.00591738 |
| RAET1G | -1.714 | 0.003667463 |
| SLC22A23 | -1.716 | 0.009128399 |
| DFNA5 | -1.716 | 0.02552169 |
| SCPEP1 | -1.718 | 0.016095006 |
| TCN1 | -1.718 | 0.000655432 |
| SEPT2 | -1.722 | 0.00351111 |
| DLL1 | -1.723 | 0.02632035 |
| GSTM2 | -1.724 | 0.002707227 |
| DST | -1.727 | 0.003477723 |
| TGFBR3 | -1.727 | 0.012395954 |
| ALDH3A1 | -1.728 | 0.000677157 |
| LYPD3 | -1.731 | 0.023340182 |
| IGFBP5 | -1.731 | 0.004561891 |
| MYLIP | -1.733 | 0.009577782 |
| LOC148915 | -1.733 | 0.003783873 |
| LY6D | -1.734 | 0.006059611 |
| PRODH | -1.740 | 0.002788915 |
| NAB1 | -1.741 | 0.020238685 |
| NUSAP1 | -1.743 | 0.013568718 |
| TOP2A | -1.754 | 0.040610854 |
| S1PR5 | -1.755 | 0.003719459 |
| RAP2C | -1.755 | 0.008278198 |
| TYMS | -1.755 | 0.034168225 |
| HMGN2 | -1.757 | 0.016140679 |
| SAA2 | -1.762 | 0.015362809 |
| CYP3A5 | -1.766 | 0.001075092 |
| IMPA2 | -1.768 | 0.024638865 |
| HMGB2 | -1.769 | 0.002783633 |
| KLHDC8B | -1.769 | 0.00417045 |
| IFITM1 | -1.773 | 0.014987472 |
| LOC728910 | -1.773 | 0.011092697 |
| CDKN2B | -1.775 | 0.004931139 |
| IGFL1 | -1.775 | 0.000524652 |
| IGFL3 | -1.777 | 0.00470997 |
| AKR1B15 | -1.780 | 0.004520438 |
| UBE2C | -1.784 | 0.016262289 |
| FST | -1.793 | 0.008018727 |
| OSTM1 | -1.793 | 0.015502544 |
| SPSB1 | -1.797 | 0.009459443 |
| CEBPD | -1.799 | 0.00300373 |
| GSDMC | -1.799 | 0.003937999 |
| VANGL2 | -1.801 | 0.008851354 |
| CA12 | -1.802 | 0.002930695 |
| CTSK | -1.803 | 0.014210676 |
| TP53INP1 | -1.809 | 0.004020731 |
| NOTCH1 | -1.809 | 0.001650692 |
| DEGS1 | -1.813 | 0.026036553 |
| MFAP5 | -1.814 | 0.006929549 |
| ZC3H12A | -1.820 | 0.007349947 |
| RHOB | -1.823 | 0.01172817 |
| TP73L | -1.823 | 0.000447004 |
| TSPAN7 | -1.827 | 0.003727208 |
| DSC3 | -1.828 | 0.039260391 |
| IL33 | -1.829 | 0.003654302 |
| IGFBP3 | -1.829 | 0.015155636 |
| PTMA | -1.830 | 0.00146582 |
| ALDH3B2 | -1.830 | 0.021191926 |
| VIPR1 | -1.845 | 0.001562044 |
| DAPL1 | -1.846 | 0.0082389 |
| FAM89A | -1.850 | 0.005503977 |
| MAF | -1.860 | 0.00234825 |
| PDPN | -1.866 | 0.007313251 |
| COL7A1 | -1.866 | 0.003926618 |
| C6ORF85 | -1.869 | 0.000561131 |
| APCDD1 | -1.870 | 0.018136686 |
| CDCA7 | -1.873 | 0.001501094 |
| LOC643287 | -1.883 | 0.002204298 |
| OLFML2A | -1.884 | 0.013469265 |
| CXCR7 | -1.885 | 0.00698622 |
| FAM83C | -1.891 | 0.006394721 |
| TXNIP | -1.893 | 0.029417584 |
| SERPINB3 | -1.900 | 0.001188001 |
| SCOC | -1.907 | 0.020650176 |
| FBXO32 | -1.915 | 0.035528021 |
| SULF2 | -1.920 | 0.005835605 |
| ZNF323 | -1.927 | 0.003568777 |
| HSPBL2 | -1.933 | 0.004660717 |
| ANKRD35 | -1.935 | 4.77968E-05 |
| TP53AIP1 | -1.935 | 0.000494278 |
| VSNL1 | -1.938 | 0.003204687 |
| MYLK | -1.944 | 0.029307564 |
| C7ORF10 | -1.944 | 0.00113744 |
| C20ORF11 | -1.957 | 0.000915111 |
| RDH12 | -1.966 | 0.000691933 |
| ALDH3B2 | -1.986 | 0.000506988 |
| LOC648585 | -1.987 | 0.005178755 |
| TRIM22 | -1.998 | 0.002953457 |
| IGFBP3 | -2.000 | 0.020404799 |
| CA2 | -2.019 | 0.002972902 |
| CFB | -2.021 | 0.015667513 |
| SAA1 | -2.027 | 0.013871958 |
| PIK3IP1 | -2.041 | 0.001738904 |
| FABP5L2 | -2.048 | 0.002767417 |
| BBOX1 | -2.050 | 0.002219476 |
| PTGS2 | -2.062 | 0.013876374 |
| NIPAL4 | -2.070 | 0.000787087 |
| BTG2 | -2.095 | 0.000228994 |
| CLCA4 | -2.098 | 0.000253153 |
| FBXO32 | -2.103 | 0.014835296 |
| CA2 | -2.124 | 0.001155114 |
| ADM | -2.144 | 0.015230488 |
| PAMR1 | -2.161 | 0.003683613 |
| CA12 | -2.175 | 0.004494245 |
| GPNMB | -2.177 | 0.004328861 |
| TNFSF10 | -2.179 | 0.000912182 |
| ETS2 | -2.186 | 0.000473537 |
| GJA1 | -2.210 | 0.005848364 |
| LOC648585 | -2.221 | 0.001960778 |
| IFNGR1 | -2.234 | 0.005400711 |
| DSG1 | -2.274 | 0.005031578 |
| PPP2R2B | -2.317 | 0.0055417 |
| GPNMB | -2.326 | 0.005959394 |
| IL1F9 | -2.343 | 0.000331001 |
| LOC642956 | -2.346 | 0.006201712 |
| TP53INP1 | -2.351 | 0.0038008 |
| CXCR7 | -2.360 | 0.005267819 |
| MX1 | -2.395 | 0.021072166 |
| HSPB3 | -2.416 | 0.00535114 |
| LOC387934 | -2.419 | 0.003992516 |
| GPX2 | -2.445 | 0.000424331 |
| FABP5 | -2.475 | 0.000321479 |
| MAFB | -2.540 | 0.002695534 |
| FABP5L2 | -2.568 | 0.003382583 |
| KRT6C | -2.603 | 0.00454904 |
| AKR1C3 | -2.607 | 0.000769294 |
| FABP5 | -2.631 | 0.002752444 |
| KRT77 | -2.883 | 0.000353512 |
| S100A8 | -2.889 | 0.001805361 |
| AKR1B10 | -2.940 | 0.004732517 |
| AKR1C4 | -3.017 | 0.000797445 |
| AKR1C2 | -3.140 | 0.000756586 |
| KRT10 | -3.332 | 0.000536988 |
| C10ORF99 | -3.416 | 0.002449894 |
| DSC1 | -3.698 | 9.03566E-05 |
| DSC1 | -3.841 | 4.47723E-05 |
| KRT1 | -3.946 | 0.000216355 |
