# Supplementary material for: Mutations in TSPEAR, Encoding a Regulator of Notch Signaling, Affect Tooth and Hair Follicle Morphogenesis
Source: PLoS Genet. 2016 Oct 13;12(10):e1006369. doi: 10.1371/journal.pgen.1006369 (PMC5065119; doi:10.1371/journal.pgen.1006369)
Supplement: S3 Table — (Global gene expression data from human primary KCs transfected with control siRNA or TSPEAR siRNA (pooled from three independent experiments) were analyzed for upstream regulators using the IPA software). (DOCX) [file pgen.1006369.s003.docx]

**S3 Table. Ingenuity Pathway Analysis (IPA) of upstream regulators**

(Global gene expression data from human primary KCs transfected with control siRNA or *TSPEAR* siRNA (pooled from three independent experiments) were analyzed for upstream regulators using the IPA software)

| Upstream regulator | Gene Exp. [log2] | Enrichment analysis p-value | Target molecules in dataset |
| --- | --- | --- | --- |
| TP63 | -0.867 | 9.20E-10 | ADM,CCNA2,CDKN2B,DLL1,FST,GPX2,IGFBP3,KRT1,KRT10,NT5E,RAC2,S100A8,TP53AIP1,TP63 |
| IL1B | -0.642 | 1.40E-06 | ADAM8,BTG2,CEBPD,FABP5,FST,IFNGR1,IGFBP5,MUC1,PTGS2,S100A8,SAA1,TXNIP,ZC3H12A |
| MGEA5 | -0.608 | 1.97E-05 | ADAM19,CDCA7,CDKN2B,CLDN7,DST,IGFBP3,IGFBP5,KRT6C,KRT81,RHOB,TGFBR3 |
| NOTCH1 | -0.855 | 6.98E-05 | CDCA7,DLL1,IGFBP3,TGFBR3,TP63 |
| ID3 | -0.657 | 6.98E-05 | ETS2,IFNGR1,MAF,NOTCH1,PIK3IP1,TGFBR3,TNFSF10 |
| TGM2 | 1.111 | 1.17E-04 | BTG2,CA2,CDCA7,GJA1,MAFB,S100A8,SLC20A1,TRIM22 |
| RXRA | -0.646 | 1.79E-03 | AKR1C1/AKR1C2,CCNA2,CEBPD,CYP3A5,NAV2 |
| TXNIP | -0.921 | 1.90E-03 | CCNA2,PTGS2,TXNIP |
| DSP | -0.692 | 1.91E-03 | DSC2,DSC3 |
| OSTM1 | -0.842 | 2.33E-03 | CTSK,MAFB |
| S100A8 | -1.531 | 3.94E-03 | IL33,LOC102724788/PRODH,PIK3IP1,S100A8,TXNIP |
| ACKR3 | -1.239 | 4.36E-03 | ADM,HBEGF |
| SMARCA4 | -0.600 | 5.35E-03 | ANO1,CDKN2B,COL7A1,HS3ST1,IFITM1,IGFBP5,MUC1,MYLK,RAC2 |
| NAB1 | -0.800 | 6.65E-03 | IFNGR1 |
| PRC1 | -0.730 | 6.65E-03 | PTGS2 |
| PKP2 | 0.591 | 6.65E-03 | GJA1 |
| NFKBIA | -0.592 | 8.57E-03 | PTGS2,S100A8,TNFSF10 |
| PTGS2 | -1.044 | 8.78E-03 | PTGS2,PTMA,TGM2,TNFSF10 |
