# Supplementary material for: Mutations in TSPEAR, Encoding a Regulator of Notch Signaling, Affect Tooth and Hair Follicle Morphogenesis
Source: PLoS Genet. 2016 Oct 13;12(10):e1006369. doi: 10.1371/journal.pgen.1006369 (PMC5065119; doi:10.1371/journal.pgen.1006369)
Supplement: S4 Table — (DOCX) [file pgen.1006369.s004.docx]

**S4 Table. Exome sequencing data**

| **Family A** | **IV-4** | **III-7** | **IV-3** | **III-5** |
| --- | --- | --- | --- | --- |
| Average read depth | 108.9 | 108.7 | 134.2 | 125.2 |
| % of exome >1x | 99.7 | 99.8 | 99.7 | 99.8 |
| % of exome >5x | 99.3 | 99.4 | 99.3 | 99.4 |
| % of exome >10x | 98.7 | 98.8 | 98.9 | 98.9 |
| % of exome >20x | 97.3 | 97.3 | 98 | 97.8 |
|  |  |  |  |  |
| **Family C** | **I-1** | **I-2** | **II-1** |  |
| Average read depth | 107.2 | 105.1 | 103.7 |  |
| % of exome >1x | 99.8 | 99.6 | 99.6 |  |
| % of exome >5x | 99.4 | 99.2 | 99.2 |  |
| % of exome >10x | 98.9 | 98.7 | 98.6 |  |
| % of exome >20x | 97.2 | 97.3 | 97.1 |  |
