# Supplementary material for: Mutations in TSPEAR, Encoding a Regulator of Notch Signaling, Affect Tooth and Hair Follicle Morphogenesis
Source: PLoS Genet. 2016 Oct 13;12(10):e1006369. doi: 10.1371/journal.pgen.1006369 (PMC5065119; doi:10.1371/journal.pgen.1006369)
Supplement: S5 Table — (DOCX) [file pgen.1006369.s005.docx]

**S5 Table. Oligonucleotide sequences used for DNA sequencing**

| **Gene** | **Exon** | **Forward** | **Reverse** | **Expected size (bp)** |
| --- | --- | --- | --- | --- |
| ***TP63*** | 1 | TCCCGGCTTTATATCTATATATAC | GACACATTCATAATACACAAGGCAC | 211  300 |
|  | 2 | TCCACTTGGGTTTTCATGATAGAG | GTAAGCAATATTTTGACCACCCAC | 300 |
|  | 3 | GCTTGTTGTTAACAACAGCATG | GAAAAGACAGGTTTAACAGAGC | 281 |
|  | 3b | AGAGAAAGAGAGAGAGGGACT | GCCACCACAGAAAACATTAAC | 337 |
|  | 4 | GATCCGTGGCTTCAGCGG | AAGCCCATCCTTGGACTTGG | 354 |
|  | 4b | CGGGACACTGACTATCAGAAC | CGTAATCCCAGCATTTCAGG | 473 |
|  | 5 | GTTGGTTCTCTCCTTCCTTTC | GCCCACAGAATCTTGACCTTC | 291 |
|  | 6 | CCACCAACATCCTGTTCATGC | GTTCTCTCAAGTCTACTCAGTCC | 267 |
|  | 7 | GGGAAGAACTGAGAAGGAACAAC | CAGCCACGATTTCACTTTGCC | 253 |
|  | 8 | AAGTGGTAGATCTTCAGGGGAC | TCCAATATCACCCCATTCTC | 260 |
|  | 9 | GCTTTAGAAGTGTTCCCAGG | ACACCTCCTTTCCCATTGTC | 237 |
|  | 10 | TGAGGATTGACCACACTTCTAAC | CATCAATCACCCTATTGCTGATC | 287 |
|  | 11 | TGTTGAAAATCAATAGTCCCCAC | TCCCCATCACAGAGTCTTGTC | 254 |
|  | 12 | CAAGATGGACCACTGGGATG | TTTATGGACTATAACAGTATCCGC | 294 |
|  | 13 | CTTATCTCGCCAATGCAGTTGG | AACTACAAGGCGGTTGTCATCAG | 240 |
|  | 14 | TGCTGTGGACTAAATGTCCG | AAGCAGGAGTGCTTTTAGGG | 449 |
|  | 15 | GATGAAGTCCTAGGCCTTC | GGAAATACAACACACACACT | 205 |
| ***TSPEAR*** | 1 | AACCAGAAGCTTCCCCACTG | CCATCTCCACAGGGTGCTAC | 427 |
|  | 2 | TATGTAAAGTGCCTGCCACG | GAGAAACCCAGAGAGTCCTTG | 445 |
|  | 3 | TCTCACCACCTGTGCTCATC | CACCTGTTCTCGCCAATGTC | 774 |
|  | 4 | GCTTAGTGAGGGGCTTGTTC | AGCACGTCCAAGGATCTGTC | 283 |
|  | 5 | TTAGATTCCATCCACCCGAG | GAAGGGCAGGATGACACTGG | 498 |
|  | 6 | TAGGCCAGGTTCTCAATTCC | AGGTCATTCAGAGGTGGCTG | 425 |
|  | 7 | CAGGAGTCGTGGCTTGTGAC | TCCTTTACCTGCAGAATCAGTG | 432 |
|  | 8 | CTGGAGAGAGATGCTGATGTTC | GTGCTGCATGTGGCTTATTG | 394 |
|  | 9 | GGTAAGAAAGGATGTCCCCAG | AGAGCAGCACTAGGTTTGGC | 498 |
|  | 10 | CTGCTCACTCACACCTCTGC | CCCAGGCCAGGAAAGTCC | 592 |
|  | 11 | GTAGCTTCTGGCCAATCCCC | GAAGCAAGGCTCTGGGAGG | 219 |
|  | 12 | GGATGGAAGAGGCTCAGATG | GAGGTGGATGGATGTCCCTG | 456 |
| ***WNT10A*** | 1 | CCCTGTGCCAGGAGGTGCC | TCTACCCCAGCAAGAGCATCAG | 441 |
|  | 2 | TTGGGACAGAGTGTGTGTTGTT | TCTGAGGTGGAGATGCTGGAT | 399 |
|  | 3 | TCCAGCCGTCCAGAAGC | CAAGGGAAAGTGCCCAGC | 518 |
|  | 4 | CTGGAGAATGGGGTGTCAAGGC | CAGAAGAGAGGTAGGCCAGGGC | 675 |
