# Supplementary material for: Mutations in TSPEAR, Encoding a Regulator of Notch Signaling, Affect Tooth and Hair Follicle Morphogenesis
Source: PLoS Genet. 2016 Oct 13;12(10):e1006369. doi: 10.1371/journal.pgen.1006369 (PMC5065119; doi:10.1371/journal.pgen.1006369)
Supplement: S6 Table — (DOCX) [file pgen.1006369.s006.docx]

**S6 Table. Oligonucleotide sequences used for qRT-PCR**

| **Gene** | **Forward** | **Reverse** | **Expected size** |
| --- | --- | --- | --- |
| DLX3 | CTCGCCCAAGTCGGAATATAC | GCTTCCCATTCACCATGC | 137 |
| DSG1 | CCCTCCAGTGTTTTCAATGGC | AATTGTTCGGTTCATCTGCG | 110 |
| *GAPDH* | GAGTCAACGGATTTGGTCGT | GACAAGCTTCCCGTTCTCAGCC | 185 |
| GATA3 | GTCCTGTGCGAACTGTCAG | CTGGTCTGGATGCCTTCC | 147 |
| GJA1 | ACATGAATTACAGCCACTAGCC | ACAATTGAGTGGAATCTTGATGC | 137 |
| HBEGF | CCTATGACCACACAACCATCC | CATGCCCAACTTCACTTTCTC | 146 |
| HES1 | CACTGATTTTGGATGCTCTGAAG | CACTTGGGTCTGTGCTCAG | 141 |
| HEY1 | AGGTGGAGAAGGAGAGTGC | AACTGTTATTGATCCGGTCTCG | 147 |
| HEYL | TTGAGAAACAGGGCTCTTCC | ACTCCCGAAAACCAATGCTC | 150 |
| IGFBP3 | ACTGAATCACCTGAAGTTCCTC | CCCATACTTATCCACACACCAG | 148 |
| IGFBP4 | AAGGCGTGTGCATGGAG | AATTTTGGCGAAGTGCTTCTG | 149 |
| KRT1 | TGAATTTGTGACCATCAAGAAGGA | TCTGCTTGGTAGAGTGCTGTAAGG | 120 |
| *KRT10* | GGAAGAATCAAACTATGAGCTG | ATTGTCGATCTGAAGCAGG | 178 |
| NOTCH1 | CTCAACATCCCCTACAAGATCG | GAACAGAAGCACAAAGGCG | 111 |
| TGFB2 | CTGTGGATGACCTGGCTAAC | CATTTCCCAGAGCACCAGAG | 146 |
| TP63 | TTCGGACAGTACAAAGAACGG | GCATTTCATAAGTCTCACGGC | 149 |
| WNT10a | CTCCTGTTCTTCCTACTGCTG | CACTGTGTTGGCATTGAGC | 108 |
| WNT4 | GTGCCAGTACCAGTTCCG | CACACCTGCCGAAGAGATG | 130 |
