# Supplementary material for: Mutations in TSPEAR, Encoding a Regulator of Notch Signaling, Affect Tooth and Hair Follicle Morphogenesis
Source: PLoS Genet. 2016 Oct 13;12(10):e1006369. doi: 10.1371/journal.pgen.1006369 (PMC5065119; doi:10.1371/journal.pgen.1006369)
Supplement: S1 Fig — TSPEAR mRNA expression in human primary KCs transfected with control siRNA or TSPEAR siRNA was ascertained using qRT-PCR. Results are expressed as percentage of gene expression in primary KCs cells transfected with TSPEAR-specific siRNA relative to gene expression in siRNA control-transfected cells ± standard error (two sided t-test: **p<0.01). Results are normalized to GAPDH RNA levels. (DOCX) [file pgen.1006369.s007.docx]

**S1 Figure. TSPEAR mRNA expression in siRNA treated keratinocytes**

**

**
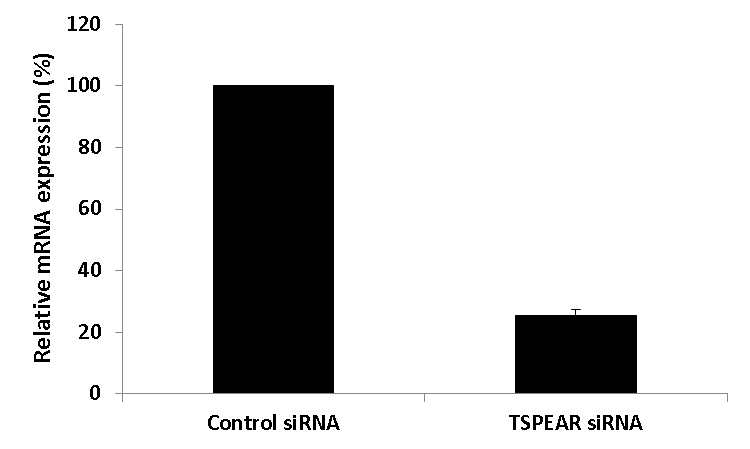
**

*TSPEAR* mRNA expression in human primary KCs transfected with control siRNA or *TSPEAR* siRNA was ascertained using qRT-PCR. Results are expressed as percentage of gene expression in primary KCs cells transfected with *TSPEAR*-specific siRNA relative to gene expression in siRNA control-transfected cells + standard error (two sided t-test: **p<0.01). Results are normalized to *GAPDH* RNA levels.
