# Supplementary material for: Mutations in TSPEAR, Encoding a Regulator of Notch Signaling, Affect Tooth and Hair Follicle Morphogenesis
Source: PLoS Genet. 2016 Oct 13;12(10):e1006369. doi: 10.1371/journal.pgen.1006369 (PMC5065119; doi:10.1371/journal.pgen.1006369)
Supplement: S2 Fig — (a) TSPEAR mRNA expression was assessed in mouse enamel organ. RNA was isolated from the enamel organ of four mice at postnatal day 10 and analyzed by RNA-seq. RNA-seq signal files were loaded into IGV (mm9) for peak visualization. The IGV track in the upper panel shows the peaks corresponding to exons 4 to 7 of Tspear. Note that Tspear is not annotated in IGV. Therefore we aligned the track to the corresponding region in UCSC genome browser (lower panel) where Tspear is annotated; (b) Immununohistochemical staining of longitudinal sections of rat mandibular incisors at P10 using anti-KRT5 (red) and anti-TSPEAR (green) antibodies. Nuclei are stained with DAPI (blue). TSPEAR was detected at the secretion front of ameloblasts at the secretory stage and persisted in the enamel matrix during the early maturation stages (white arrowheads). No staining was not detected at the late maturation stage. KRT5 was detected both in ameloblasts (above the white dotted lines) and in the papillary layer (below the white dotted lines). (DOCX) [file pgen.1006369.s008.docx]

**S2 Figure. Tspear expression in the enamel organ**


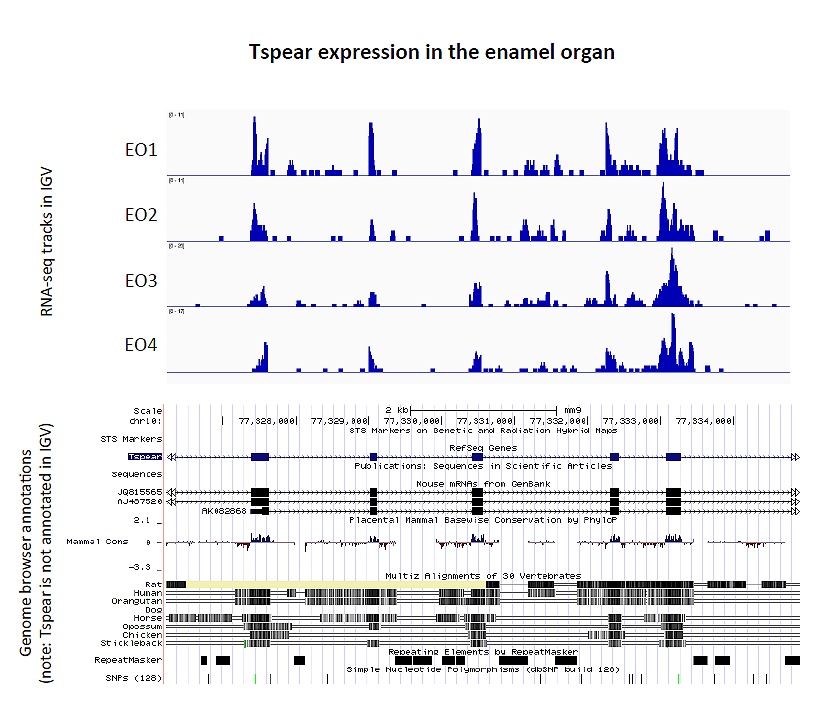


a


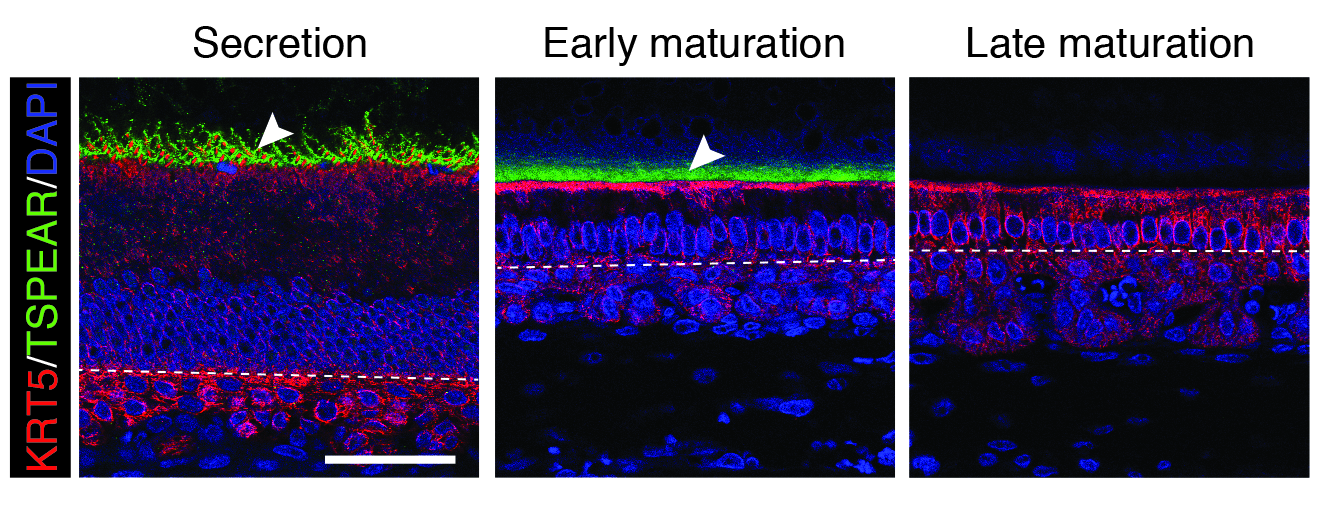


b

(a) TSPEAR mRNA expression was assessed in mouse enamel organ. RNA was isolated from the enamel organ of four mice at postnatal day 10 and analyzed by RNA-seq. RNA-seq signal files were loaded into IGV (mm9) for peak visualization. The IGV track in the upper panel shows the peaks corresponding to exons 4 to 7 of Tspear. Note that Tspear is not annotated in IGV. Therefore we aligned the track to the corresponding region in UCSC genome browser (lower panel) where Tspear is annotated; (b) Immununohistochemical staining of longitudinal sections of rat mandibular incisors at P10 using anti-KRT5 (red) and anti-TSPEAR (green) antibodies. Nuclei are stained with DAPI (blue). TSPEAR was detected at the secretion front of ameloblasts at the secretory stage and persisted in the enamel matrix during the early maturation stages (white arrowheads). No staining was not detected at the late maturation stage. KRT5 was detected both in ameloblasts (above the white dotted lines) and in the papillary layer (below the white dotted lines).
